# Experientially-grounded and distributional semantic vectors uncover dissociable representations of conceptual categories

**DOI:** 10.1080/23273798.2023.2232481

**Published:** 2023-07-12

**Authors:** Francesca Carota, Hamed Nili, Nikolaus Kriegeskorte, Friedemann Pulvermüller

**Affiliations:** a Max Planck Institute for Psycholinguistics, Nijmegen, The Netherlands; b Donders Institute for Cognitive Neuroscience, Radboud University, Nijmegen, The Netherlands; c Cognition and Brain Sciences Unit, Medical Research Council, Cambridge, UK; d Department of Experimental Psychology, University of Oxford, Oxford, UK; e MRC Cognition and Brain Sciences Unit, University of Cambridge, Cambridge, UK; f Department of Psychology, Zuckerman Mind Brain Behavior Institute, Columbia University, New York, NY, USA; g Department of Neuroscience, Zuckerman Mind Brain Behavior Institute, Columbia University, New York, NY, USA; h Department of Electrical Engineering, Zuckerman Mind Brain Behavior Institute, Columbia University, New York, NY, USA; i Brain Language Laboratory, Department of Philosophy and Humanities, WE4, Freie Universität Berlin, Berlin, Germany; j School of Mind and Brain, Humboldt Universität zu Berlin, Berlin, Germany; k Cluster of Excellence “Matters of Activity”, Humboldt Universität zu Berlin, Berlin, Germany; l Einstein Center for Neuroscience Berlin, Berlin, Germany

**Keywords:** Experiential semantics, symbol grounding, distributional statistics, language network, RSA, fMRI

## Abstract

Neuronal populations code similar concepts by similar activity patterns across the human brain's semantic networks. However, it is unclear to what extent such meaning-to-symbol mapping reflects distributional statistics, or experiential information grounded in sensorimotor and emotional knowledge. We asked whether integrating distributional and experiential data better distinguished conceptual categories than each method taken separately. We examined the similarity structure of fMRI patterns elicited by visually presented action- and object-related words using representational similarity analysis (RSA). We found that the distributional and experiential/integrative models respectively mapped the high-dimensional semantic space in left inferior frontal, anterior temporal, and in left precentral, posterior inferior/middle temporal cortex. Furthermore, results from model comparisons uncovered category-specific similarity patterns, as both distributional and experiential models matched the similarity patterns for action concepts in left fronto-temporal cortex, whilst the experiential/integrative (but not distributional) models matched the similarity patterns for object concepts in left fusiform and angular gyrus.

## Introduction

Recent brain imaging research taking advantage of the information carried by population codes (e.g. neurones or voxels) in the brain has demonstrated that similar concepts and word meanings are represented by similar activity patterns (e.g. Carlson et al., 2014; Carota et al., 2017, 2021; Devereux et al., 2013; Fernandino et al., 2022; Kriegeskorte et al., 2008; Liuzzi et al., 2017) and that the statistical distributions of words, as captured by co-occurrence frequency vectors (Landauer & Dumais, 1997; Mikolov et al., 2013), relate to such representational patterns in the language networks (e.g. Carlson et al., 2014; Carota et al., 2017, 2021; Fu et al., 2022; Pereira et al., 2018; Wang et al., 2020; Xu et al., 2018). Distributional statistics, capturing the meanings of words and concepts by the company they take, have been proposed to provide a basis for language comprehension (e.g. Landauer, 1998; Landauer & Dumais, 1997).

However, distributional data in isolation fail to provide a cognitively realistic model of semantic knowledge (e.g. Harnad, 1990), because they do not clarify the links between word symbols and “the world” – the domain of referential semantics. Symbols become meaningful if anchored, or *grounded*, in the experiences of perceptions and actions that link them up with their extralinguistic referents (see Barsalou, 1999, 2008, 2017; Gallese & Lakoff, 2005; Glenberg & Robertson, 2000; Harnad, 2012; Marino et al., 2012; Pulvermüller, 1999, 2018) – for example, the object-related word *peach* with grounded semantic features such as [ + ROUND], [ + YELLOW] and the action word *hike* with [ + LEGS], [ + MOVEMENT]. Such experiential attributes can be used to efficiently code conceptual and semantic structure (Binder et al., 2016). They are reflected in reaction times during semantic judgments and lexical decision tasks (Andrews et al., 2014; Barsalou, 1999; Barsalou et al., 2008; Kousta et al., 2011; Paivio, 1971; Vigliocco et al., 2014; Zwaan, 2014), and likewise in the spatial distributions of brain activity patterns elicited by different semantic categories and indeed individual symbols (Fernandino et al., 2016, 2022; Hauk et al., 2004; Martin, 2007; Pulvermüller, 2018). Psycholinguistic theory holds that such experiential knowledge is linguistically encoded as a function of language use and task at hand (Symbol Interdependency hypothesis: Louwerse, 2008; 2018; 2021), so that an increase in perceptual simulation allows for richer and more detailed conceptual representations. Corroborating this view, recent behavioural results suggest that the sensorimotor properties of concepts enable a more precise specification of semantic categories, resulting into a best mapping of semantic similarities among concepts, as compared to distributional statistics *per se* (e.g.: Binder et al., 2016). As suggested by Carota et al. (2021), experiential properties are intercorrelated to a different degree with distributional data and with the hierarchical/taxonomic structure. The latter define categorical similarity between words, e.g. *peach* and *plum,* on their distance from their shared superordinate node, in this case “fruit”, and their shared overarching node, here “object”. Likewise, semantic dissimilarity, e.g. between *swan* and *hike,* depends on distance from the superordinate nodes “animal”/“object”, and “action”, respectively (see Carota et al., 2021 for discussion). In turn, distributional models coding word meaning in terms of linguistic contexts, based on word co-occurrences in texts, tend to produce semantic clusters that align with linguistic (or visual) context (Baroni et al., 2014; Bonner & Epstein, 2021; Crutch et al., 2013; Popham et al., 2021).

The goal of the present study is to investigate the commonalities and differences between grounded experiential and distributional semantic similarity mappings, and their integration.

It is well known that concepts and word meanings belonging to different categories are processed differently in the human brain; for example, action-related words and object words activate different sets of brain areas (Carota et al. 2012; Damasio et al., 1996; Hauk et al., 2004). The differences in activation patterns crucially depend on the different sensory and motor attributes that are pertinent for defining a given concept (Carota et al., 2017; Fernandino et al., 2016, 2022; Pulvermüller et al., 2009).

Previous work indicated that experiential semantic attributes are better reflected in the representational patterns of the brain's “semantic network” than distributional information not only in these unimodal regions, but also in regions in multimodal association cortex (Fernandino et al., 2022), which integrate unimodal sensory and motor features. Different accounts have ascribed an integration role to anterior temporal cortex (aTL: Lambon Ralph et al., 2017; Patterson et al., 2007), middle temporal gyrus (MTG: Hickok, 2014; Turken & Dronkers, 2011), inferior temporal gyrus (ITG, Price, 2000), angular gyrus (AG: Binder et al., 2009; Fernandino et al., 2016; Geschwind, 1965), or a range of fronto-parieto-temporal “connector hub” areas (Garagnani & Pulvermüller, 2016). Some of these multimodal integration regions are also good candidates for encoding distributional information, as they are part of the thematic network (Mirman et al., 2017; Wang et al., 2020; Xu et al., 2018), as well as intercorrelated experiential semantic properties flexibly mediated in multiple cortical regions (e.g. Reilly et al., 2016). In particular, recent evidence (Carota et al., 2021) suggested that the angular gyrus is important to interface distributional representations of concepts and their action-related properties, whereas anterior aspects of left inferior frontal gyrus (LIFG, BA 45-47), known to enable semantic combinatorial operations unifying simpler meaningful units (e.g. words) into larger semantic structures (e.g. sentences) (e.g. Hagoort, 2005, 2015, 2019), may primarily support representational similarity based on the distributional statistics (Carota et al., 2021). The relevance of this region for semantic combinatorics has been explained as rooted in its executive – rather than representational – functions (e.g. Hoffman et al., 2018). Intriguingly though, some studies indicate that also category-preferential visual and motor regions may take a role in representing distributional similarities for specific semantic word categories (Bonner & Epstein, 2021; Carlson et al., 2014; Carota et al., 2017; Hauk et al., 2004; Liuzzi et al., 2020; Mitchell et al., 2008; Popham et al., 2021; Pulvermüller, 1999). For instance, distributional semantic similarities among words were indexed in posterior inferior temporal cortices (e.g. Carlson et al., 2014), for which previous studies demonstrated category selectivity for different types of objects and nouns (e.g. places, foods, tools, and animals) (Mitchell et al., 2008; Mitchell and Cusack, 2016), and in prefrontal and posterior middle temporal regions, in which earlier work demonstrated the presence of representational similarities linked to rated experiential properties (particularly action-relatedness), and hierarchical taxonomies for action-related concepts (Carota et al., 2021 for detailed discussion). Reflections of the distributional links among action-related words has been also found in the dorsal region of the LIFG (BA44-45) (Carota et al., 2017), where the similarity in action-relatedness of the stimuli was also highly correlated with the similarity structure of the brain activity patterns (Carota et al., 2021).

These results lead to the hypothesis that qualitatively distinct sources of semantic information may be redundantly represented both in higher-order association cortex and in lower-level category-preferential regions depending on category information.

Here, we asked to what extent

1. Experientially-grounded and distributional semantic representational similarities are differently mapped in modality-preferential and multimodal areas and a multidimensional vector space combining grounded semantic information (based on human ratings of semantic properties) and distributional statistics (modelled using GloVe: Pennington et al., 2014) captured the semantic similarity structure of word representations better, or more completely, than each of these models on its own;
2. higher-order regions (e.g. LIFG, aTL, MTG/ITG, AG) and lower-level unimodal regions best indexed the neurometabolic correlates of the semantic similarities between concepts and whether such representational similarity mapping differed spatially between different word types (action- and object-related words).

To elucidate the relative contribution of experiential and distributional information, as well as their combination, to the mapping of the brain's multidimensional semantic space, we reanalysed an existing dataset of recorded fMRI responses (Carota et al., 2021), which had previously been scrutinised using Representational Similarity Analysis (RSA) (Kriegeskorte et al., 2008).

## Materials and methods

### Participants

Twenty-three healthy volunteers participated in the study. All participants were healthy right-handed, monolingual English native speakers, aged on average 29 years (SE = 2.8). All participants gave their informed consent to take part in the study and were remunerated for their time. Ethical approval was obtained from the Cambridge Psychology Research Ethics Committee.

### Experimental procedure

*Stimuli*. Ninety-six words, sixteen from each individual category of leg-, arm-, face-related actions and tool-, animal-, food-related objects, were selected based on established semantic ratings (Carota et al., 2012, 2017, 2021; Hauk et al., 2008; Pulvermüller, 1999). Stimulus word groups were matched for a range of psycholinguistic properties, including word length (counted in number of letters), letter bigram and trigram frequency, logarithmic word frequency, number of orthographic neighbours, and standardised lexical frequency, while differing in imageability, concreteness, and action-relatedness (see Table 1).

**Table 1.** Psycholinguistic properties and semantic ratings are shown for each word sub-category, as well as for the categories of action- and object-related words. Means and standard errors (in brackets) are reported for each word category, along with results of an ANOVA comparing ratings between word groups.

	*Arm*	*Leg*	*Face*	*Animal nouns*	*Food nouns*	*Tool nouns*	Main effect of word-type (F)	*Action verbs*	*Object nouns*	Action x Object
**Length**	4.81 (.16)	4.63 (.16)	4.50 (.13)	4.69 (.15)	4.56 (.13)	4.63 (.16)	.533 *p*=.751	4.65 (.09)	4.63 (.082)	.030 *p*=.862
**Bigram freq.**	31248.64 (3138.66)	32472.14 (4035.53)	29029.40 (2887.87)	31538.82 (2985.33)	32699.44 (3939.17)	30550.20 (3422.15)	.155 *p*=.978	30916.73 (1926.37)	31596.15 (1964.98)	.061 *p*=.806
**Trigram freq.**	2475.48 (283.17)	2673.85 (367.88)	2535.13 (301.53)	2601.56 (399.53)	2825.27 (299.75)	2209.20 (300.13)	.401 *p*<.847	2561.49 (180.96)	2545.34 (193.65)	.004 *p*=.952
**No. of neighbours**	5.19 (0.91)	5.06 (0.60)	5.44 (0.84)	5.25 (0.76)	4.81 (0.79)	5.25(0.84)	.071 *p*=.996	5.23 (.45)	5.10 (.45)	.039 *p*=.845
**No. of meanings**	1.06 (0.06)	1.13 (0.09)	1.13 (0.09)	1.13 (0.09)	1.06 (0.06)	1.19 (0.10)	.333 *p*=.892	1.10 (.05)	1.13 (.05)	.101 *p*=.752
**Log. word freq.**	0.66 (0.11)	0.57 (0.13)	0.56 (0.10)	0.61 (0.11)	0.61 (0.14)	0.72 (0.12)	.250 *p*=.939	.60 (.06)	.65 (.70)	.266 *p*=.607
**Imageability**	4.47 (0.17)	4.45 (0.27)	3.97 (0.26)	6.32 (0.09)	5.48 (0.27)	5.35 (0.35)	11.969 *p*<.001	4.30 (.14)	5.72 (.16)	44.722 *p*<.0001
**Concreteness**	4.14 (0.16)	3.59 (0.19)	3.62 (0.19)	6.60 (0.08)	6.21 (0.19)	5.73 (0.21)	62.306 *p*<.001	3.78 (.11)	6.18 (.11)	250.451 *p*<.001
**Action-relatedness**	4.83 (0.23)	4.91 (0.24)	5.31 (0.25)	1.60 (0.10)	2.02 (0.30)	3.22 (0.41)	34.967 *p*<.001	5.02 (.14)	2.28 (.19)	129.953 *p*<.001
**Face-relatedness**	1.56 (0.10)	1.40 (0.08)	5.75 (0.23)	1.20 (0.07)	2.06 (0.26)	1.27 (0.10)	123.410 *p*<.001	2.90 (.31)	1.51 (.11)	18.344 *p*<.001
**Arm-relatedness**	5.68 (0.13)	1.93 (0.17)	1.33 (0.09)	1.11 (0.06)	1.37 (0.12)	2.85 (0.36)	89.026 *p*<.001	2.98 (.29)	1.78 (.17)	12.816 *p*=.001
**Body Sensation**	3.74 (0.29)	3.55 (0.22)	3.92 (0.31)	1.16 (0.07)	1.35 (0.11)	1.40 (0.16)	40.353 *p*<.001	3.74 (.16)	1.30 (.07)	28.050 *p*<.001
**Valence**	3.45 (0.27)	4.05 (0.20)	3.66 (0.31)	3.52 (0.09)	4.08 (0.13)	3.85 (0.14)	1.626 *p*=.161	3.71 (.15)	3.81 (.08)	.377 *p*=.541
**Arousal**	3.25 (0.27)	2.98 (0.20)	2.60 (0.29)	1.30 (0.21)	1.44 (0.16)	1.65 (0.19)	14.464 *p*<.001	2.94 (.15)	1.46 (.11)	65.591 *p*<.001
**Colour**	2.10 (0.27)	1.56 (0.11)	1.43 (0.18)	2.55 (0.19)	3.39 (0.37)	1.41 (0.15)	11.707 *p*<.001	1.70 (.12)	2.45 (.19)	11.543 *p*=.001
**Shape**	2.96 (0.30)	2.46 (0.21)	1.73 (0.21)	3.67 (0.20)	2.90 (0.26)	3.21 (0.21)	8.140 *p*<.001	2.38 (.16)	3.26 (.13)	18.231 *p*<.001
**Sound**				1.39 (0.90)	1.03 (0.02)	1.85 (0.34)	57.105 *p*<.001		1.42 (.12)	
**Taste/Smell**				1.93 (0.24)	4.46 (0.30)	1.11 (0.08)	4.114 *p*=.023		2.49 (.25)	
**Motion**	3.11 (0.21)	4.37 (0.10)	2.63 (0.15)	1.63 (0.07)	1.41 (0.15)	2.23 (0.30)	4.716 *p*=.014		1.75 (.12)	

Relevant values were obtained from the CELEX database (Baayen et al., 1993) and the WordSmyth Web site (www.wordsmyth.net/). 21% of the action words were lexically unambiguous verbs and the lexically ambiguous ones which could be used as nouns and verbs were in the average 14 times more frequently used as verbs than as nouns (according to the Celex database; SE 4.2). 58% of the object words were lexically unambiguous nouns and the lexically ambiguous ones which could be used as nouns and verbs were in the average 6 times more frequently used as nouns than as verbs (SE 2). The object categories exhibited a gradient in action-relatedness, as animals were not associated with action (note that no pets were included), but to visual properties, food afford the actions of mouth and hand related to eating and preparing food, and tools afforded a greater variety of hand, foot, and mouth actions. The tool concepts were still highly associated with visual properties. We included object words with differing in their action-relatedness in order to test the power of the test models used here to capture the similarity and differences between the classes of object concepts and action concepts. This was indeed a part of the experimental design we were interested in, which also differs from most of earlier studies focusing on either action or object concepts.

Strings of meaningless hash marks matched in length to the stimulus words were used as low-level baseline stimuli during 120 trials. Null events consisting of a fixation cross displayed at the centre of the screen were presented during additional 60 trials. 60 trials consisting of misspelled words to be detected by the participants throughout the experimental task were presented. These “typo” trials did not include words from any of the semantic categories from which the 96 target words were taken – so as to avoid a bias towards one of these categories – and were discarded from the analysis. Statistical comparisons were carried out between brain activation patterns elicited by matched word categories.

After the fMRI experiment, participants completed an unannounced word recognition test containing both novel distractor and experimental words. They performed above chance (average hit rate: 85% [STD: 8.3%]), indicating their attention to the words and compliance with the task. Results were used to confirm that subjects had been attentive continuously during the silent reading task.

*Experimental Design*. We adopted a rapid, periodic single trial, event-related paradigm. Fixation cross was presented at the centre of the screen whenever no stimulus was shown. Word and hash string stimuli were presented tachistoscopically, to discourage subjects from eye movements, for 100 ms. The stimulus onset asynchrony (SOA) was randomly varied between 3.5 and 4s. This design yielded overlapping, yet detectable haemodynamic responses (Kriegeskorte et al., 2008; Nili et al., 2014). The 96 stimulus words were presented in a different pseudo-random order in each of the 6 runs. Each stimulus occurred once per run (6 repetitions of each word in total). Stimuli were visually presented by means of E-Prime software (Psychology software Tools, Inc., Sharpsburg, USA, 2001) through a back-projection screen positioned in front of the scanner and viewed on a mirror placed on the head coil.

*Task*. Participants were engaged in a typo-detection task. They were given the instruction to attend to all the stimuli, to silently read the words and to understand their meaning. In addition, they were instructed to press a button with their left index finger if a misspelled word appeared at the centre of the screen. To avoid confounding by body movements and responses, all catch trials with or without manual responses to typos were excluded from any analyses.

As the study made use of data from previously published work (Carota et al., 2017, 2021), we will summarise the principal method points, and refer the reader to the original article for the details (Carota et al., 2021).

#### Imaging methods

Subjects were scanned in a Siemens 3 T Tim Trio using a head coil. Echo-planar imaging (EPI) sequence parameters were TR = 2000 msec, TE = 30msec, and flip angle = 78°. The functional images consisted of 32 slices covering the whole brain (slice thickness 3 mm, in-plane resolution 3 × 3 mm, inter-slice distance 0.75 mm).

#### Differences with earlier work

Previous work reporting on the present data set focused on related, yet different questions. A first study investigated the brain correlates of distributional semantics by evaluating brain responses averaged across semantic categories (Carota et al., 2017), whilst a second study focused on item-specific analyses of brain responses reflecting distributional semantics (using LSA methods) and hierarchical semantic structure (based on data from WordNet) (Carota et al., 2021). However, none of the previous studies explored the relative contribution of different sensorimotor attributes and distributional semantics. Here, we now ask how distributional and grounded experiential semantics are reflected in the similarity structure of word-elicited brain activation and whether a combination of both semantic approaches leads to a closer relationship between semantic similarities and similarities of the brain responses of the words. Furthermore, in the present study (but not in our previous ones), the analyses were conducted for the entire group of words, irrespective of their category, as well as for specific categories of action and object words, in order to detect representational differences between category-general groups of concepts, and specific categories of concepts. Importantly, to do this, we directly compared our three target models in a whole set of regions of interest, as well as in each region of interest individually.

#### Data analysis: general linear model for representational similarity analysis (RSA)

For multivariate RSA (Kriegeskorte et al., 2008; Nili et al., 2014), the analysis was carried out in participant native space, using realigned, unsmoothed and non-normalised functional data, which were co-registered with MPRAGE of each subject. Data were analysed using the general linear model. Response-amplitude was estimated for each voxel and for each of the 96 stimuli by performing single univariate linear model fit. Runs were concatenated along the temporal dimension. A separate haemodynamic predictor was included for each of the 96 stimulus words. As each word stimulus occurred once in each run, each of the 96 stimulus words had one distinct haemodynamic response per run, extended across all 6 runs. The response-amplitude (beta) estimate map associated with each stimulus trial across all (6) runs was converted into a t map by contrasting them against the implicit baseline in order to compute the representational dissimilarity matrices for RSA (Carota et al., 2021; Nili et al., 2014).

### Model specification

**Figure 1.**
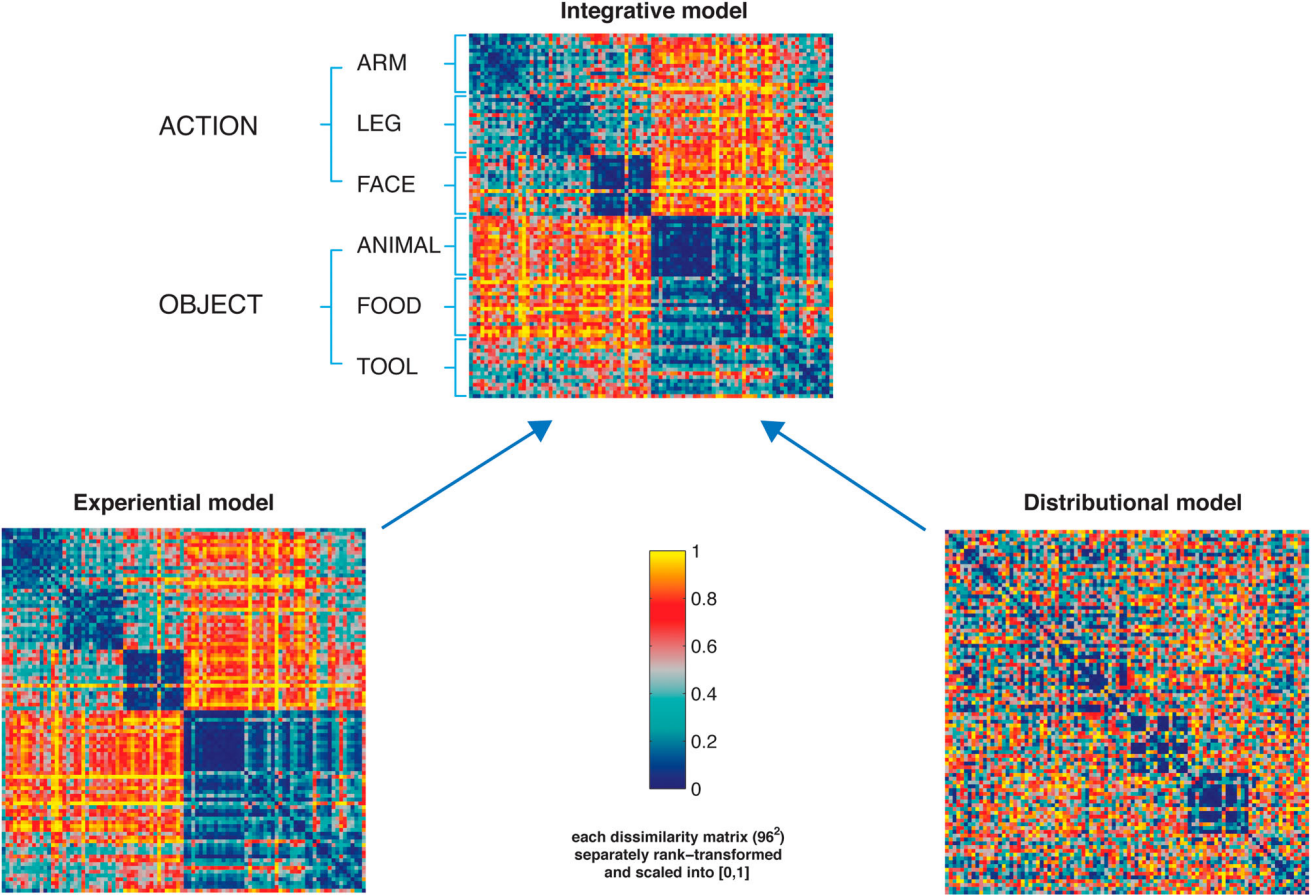
Representational dissimilarity matrices (RDM) displaying the semantic distances among the 96 stimulus words based on (a) the grounded “experiential” model reflecting human ratings (bottom left); (b) the distributional model based on GloVe (bottom right) and (c) the integrative model (top panel). The dissimilarity values from the two matrices were normalised before integration by averaging. Dark blue ( =  0) indicates maximal similarity between identity pairs and yellow ( = 1) indexes maximal dissimilarity. Each square in the model RDM shows (dis)similarities between the individual word items, which are plotted from top to bottom and from left to right – with dark blue indicating great semantic similarity or identity and red/yellow indexing dissimilarity. Therefore, the first diagonal in the matrix indicates the semantic identity of each single item with itself. Note that the integrated model at the top (see Table 2 for statistical tests) reflects a the categorial semantic structure of the stimulus vocabularies; please note the series of six blue squares form top left to bottom right representing semantic similarities within each of the six semantic subtypes. Also, the lexical categories of verbs and nouns are clearly revealed by this integrated model (big red/yellow “dissimilarity squares” at lower left and upper right). The only exception from the between-lexical category-dissimilarity is seen for tool words, which are indeed semantically associated with action verbs (for example, “knife” and “carve”/ “peel”). The sensorimotor model shows the similarities differences in lexicosemantic categories comparably. The distributional model also reflects the categorical semantic structure of the previous models to a lesser extent.

In order to test for the contribution of grounded experiential and distributional semantics on the representation of different word categories, we built up a semantic vector combining these qualitatively different properties (see Figure 1).

*Experiential Model*. The grounded experiential semantic model was constructed based on the following rated semantic properties relevant for the test words (for discussion, see Pulvermüller, 1999; and Binder et al., 2016): (1) action-relatedness (for discussion regarding action-relatedness for object concepts, see Carota et al., 2012), (2) arm-relatedness, (3) leg-relatedness, (4) face-relatedness, (5) colour, (6) shape, (7) visual-relatedness, (8) imageability, (9) body-sensation, (10) arousal, and (11) valence. The ratings were acquired in a separate study using Lime Survey (http://www.limesurvey.org LimeSurvey Project Team/Carsten Schmitz, 2013). Fifteen native speakers of English were recruited to provide ratings for each word for a number of semantic variables, covering (1) sensorimotor meaning features – including imageability, concreteness, visual-relatedness (colour, shape), body-relatedness, and action-relatedness – and (2) affective–emotional features – including arousal and valence (Bradley & Lang, 1994; Osgood et al., 1975). We administered explicit semantic ratings asking the following questions:

1. Is this word easy to imagine?
2. Is this word typically used to speak about something you can concretely experience (touch, see, hear)?
3. Is this word typically used to speak about visually perceived scenes or objects?
4. Is this word typically used to speak about colour?
5. Is this word typically used to speak about shape?
6. Is this word typically used to speak about activities you perform yourself (e.g. to move)?
7. Is this word typically used to speak about activities you perform with the face?
8. Is this word typically used to speak about activities you perform with the arm?
9. Is this word typically used to speak about activities you perform with the legs?
10. Is this word typically used to speak about something exciting (arousal)?
11. Is this word typically used to speak about negative or positive emotions (valence)?

Participants used a 1-7 Likert scale to provide their judgements. Please see Pulvermüller (1999) for further discussion.

Therefore, in the experiential model, each word was represented as n(11)-dimensional vector, where dimensions correspond to the semantic properties (see list above) and the value for each dimension was the average rating of a given property. For each word pair, we calculated the correlation distance (1-r) between the values of each of these vectors, and entered the resulting values in each cell of a Representational Dissimilarity Matrix (RDM) (Figure 1, left bottom panel). We reasoned that 1-correlation (Pearson correlation across space) could be preferred for the experiential models and the neural RDMs (Kriegeskorte et al., 2008), because it is the most commonly used measure for RDMs. To construct the distributional vector model, we adopted cosine similarity to follow the classical metric for the calculation of semantic similarity based on co-occurrence frequencies in the tradition of Latent Semantic Analysis, as described in the next paragraph. Please note that Pearson correlation and cosine similarity are closely related, differing only in the fact that Pearson correlation subtractively normalises the mean to each pattern (to 0), while both methods divisively normalise the pattern variance (to 1).

*Distributional Model*. We applied a state-of-art extension of Latent Semantic Analysis (LSA, Landauer & Dumais, 1997), GloVe (Pennington et al., 2014). Similar to LSA, the model assumes that words have similar meaning if they tend to occur, beyond the *same* textual span, in *similar* contexts. Therefore, our distributional model indexes the semantic relationships between both words which co-occur in the same texts and paragraphs (first-order co-occurrence: for example, words linked to a common event or function, e.g. *cake* and *spoon, peach* and *to peel, harp* and *to play*), and, most importantly, words which do not appear in the same text, but can co-occur in similar contexts (second order co-occurrence). For instance, although *play* and *music* may not appear in the same text, they may separately co-occur with words like *symphony, orchestra* or *recording*. Thus, the distributional model captures abstract, second-order semantic information about word meanings, reflecting statistical knowledge about their actual usage in language. The distributional model was generated from a lexical corpus, according to established methods (see Bruffaerts et al., 2013 for a discussion on the use of different types of semantic models in neuroimaging). We used the pretrained word vectors that the GloVe authors (Pennington et al., 2014) reported their highest model performance with on the word analogy and word similarity tasks. These vectors have a length of 300 dimensions and were obtained by training on 42 billion tokens of text. The Glove method is a “global log-bilinear regression model that combines the advantages of the two major model families in the literature: global matrix factorization and local context window methods” (Pennington et al., 2014). In natural language processing, global matrix factorisation is based on matrix factorisation methods from linear algebra to reduce large term frequency matrices representing the occurrence or absence of words in a document. The model is based on weighted least squares, and trains on global word-word co-occurrence counts exploiting only the nonzero elements in a word-word co-occurrence matrix, rather than on the entire sparse matrix or on individual context windows in a large corpus. The embeddings are then optimised directly, so that the dot product of two-word vectors corresponds to the log of the number of times the two words will occur near each other. For example, if the two words “apple” and “pear” occur in the context of each other, say 20 times in a 10-word window in the document corpus, then: Vector(apple). Vector(pear) = log(10). This forces the model to encode the frequency distribution of words that occur near them in a more global context.

The model comprised 300 dimensions, the number yielding the highest model performance in the semantic task (word analogy task) evaluated by the authors (Pennington et al., 2014). Semantic similarity between words was then measured as the cosine between two-word vectors: the smaller the cosine, the greater the similarity between word stimulus pairs. These values were expressed as a dissimilarity matrix (Figure 1, right bottom panel).

*Integrative (Experiential and Distributional) Model*. The third semantic model integrated rated semantic properties and distributional properties of words, and was obtained by pointwise averaging the values contained in the two model RDMs. The similarity values from the two matrices were normalised before integration by averaging. The resulting RDM is displayed in the right panel of Figure 1 (top panel). Such integrative semantic model expressed the contribution of the distributional and sensorimotor information about the test words in terms of pairwise similarity and dissimilarity relations, positing a mutual relationship between the experiential properties of two words and their co-occurrence likelihood. In particular, the smaller the dissimilarity between the set of experiential properties for two items (e.g. for the pair peach/plum: similarity in shape, “round”; action-related, “edible/manipulable”, taste, odour, etc.), the higher their likelihood to co-occur in a similar and coherent semantic context (e.g. groceries at the market, or cooking recipe). Reversely, the bigger the experiential property dissimilarity between two items (e.g. peach and jive), the smaller their likelihood to fit similar contexts.

### Whole-brain RSA searchlights

Data were extracted for each participant individually using a “sphere of information” searchlight approach (roaming sphere with 10 mm radius: Kriegeskorte et al., 2008; Nili et al., 2014; this included about 121 voxels). See Carota et al. (2021) for method details. The correlation distances (1-correlation) between the response patterns for each word paired with every other word were expressed as representational dissimilarity matrices (RDMs), which are symmetric about a diagonal of zeros (Kriegeskorte et al., 2008). These brain data RDMs were then correlated with our model RDMs (using Spearman’s rank correlation) at each brain location. FDR correction at 0.05 for multiple comparisons across voxels and number of models was applied. 10,000 permutations were used in the analysis. In order to ensure that the searchlight maps we reported did not suffer from distortion due to either the searchlight size or the detection of fewer informative voxels, we performed additional analyses testing for the relatedness of the models and the brain activity patterns in selected regions of interest, as described below.

### Analyses per regions of interest

*Definition of ROIs.* An exploratory analysis was run in hypothesis-based, pre-selected Regions of Interest (ROIs) in the left hemisphere with well-studied semantic roles in language comprehension (for reviews: Binder & Desai, 2011; Pulvermüller, 2013), as described in the introduction. These included the (1) LIFG (BA 44, BA 45 and BA47); (2) superior, middle, and inferior temporal gyrus (STG, MTG, ITG), further divided into anterior (aSTG, aMTG, aITG) and posterior (pSTG, pMTG, pITG) sections; (3) temporal pole, TP; (4) fusiform gyrus (FuG: Mion et al., 2010); (5) precentral gyrus (PCG); and (6) inferior parietal cortex (both supramarginal gyrus: SMG, and angular gyrus: AG) (see Figure 3(A)). We automatically defined these ROIs using the standard Wake Forest University (WFU) Pickatlas toolbox, which generates ROI masks in standard MNI space based on the Automated Anatomical Labelling (AAL) parcellation. In order to carry out multivariate analysis within individual-subject native space, all ROI-masks were transformed to subject native space by inverting the spatial normalisation applied during GLM analysis. See Carota et al. (2021) for method details.

#### Relatedness between fMRI patterns and models as assessed by RSA

RDMs were computed for each participant for the abovementioned ROIs and related to the model RDMs. In order to test for the sensitivity of each of our three semantic models (i.e.: experiential, distributional, and the integrative model resulting from their combination) to semantic word categories, a first analysis in the whole brain (searchlights) and in selected ROIs was performed using the 96 × 96 matrix containing all words.

#### Results from statistical model comparisons

Analyses were run to statistically compare the model-fMRI pattern fit in all the abovementioned ROIs (results are reported only for the ROIs exhibiting significant semantic similarity effects). A first analysis directly compared the performance of the distributional, experientials and integrative models focusing on the 96 × 96 RDMs containing all words in order to assess the respective successes of the models in mapping of semantic similarity in different cortical areas. A second analysis compared the performance of the distributional, experientials and integrative models based on the 48 × 48 RDMs specific to action and object words in mapping category-specific semantic similarity in different cortical areas. A third analysis focused on the dominant attributes of action and object words, known to be reflected in the corresponding action and perception systems (e.g. Hauk et al., 2008), in order to test for the replicability of more specific category-specific semantic similarity effects in our data. To this aim, for the semantic sub-spaces of action and object words, two 48 × 48 model RDMs were constructed, a first one coding for the rated action-related properties (obtained from the ratings of action-relatedness, leg-, arm-, face-relatedness), and a second one coding for the rated visual properties (colour, shape, imageability). The model-fMRI pattern comparisons were performed by computing, for each subject, non-parametric Spearman's rank correlations between model and brain activity RDMs. The two models were compared by subtracting the r-value of the correlation between the second model and the brain activity patterns (fMRI RDM) from the r-value of the correlation between the first model and the brain activity patterns (fMRI RDM). The difference in r-value across all subjects was then tested against the null hypothesis of the value 0, to test for a difference in correlation, using a 1-sided Wilcoxon signed-rank test for all comparisons (both 96 × 96 RDM and 48 × 48 RDM). *P*-values surviving FDR correction for multiple comparisons are reported (Benjamini & Hochberg, 1995).

*Discriminability of semantic categories.* We further tested the hypothesis that action and object words are respectively encoded in fronto-central and temporal regions which are engaged by the processing of grounded action-related and visual properties of words. To this aim, an ANOVA contrasted the correlation values (as obtained from RSA) between the experiential model RDMs (48 × 48) specific to action and object words and the brain activity RDMs in a hypothesis-driven sub-set of frontal and temporal regions associated with the processing of action and object word (Hauk et al., 2004). These ROIs covered (1) the pars triangularis (BA 45) and (2) and opercularis (BA 44) of the LIFG, (3) a premotor region which was non adjacent to inferior premotor cortex and BA 44 (4) anterior temporal cortex (Patterson et al., 2007), (5) fusiform gyrus, and (6) angular gyrus (Binder & Desai, 2011). We additionally performed a 2 × 2 ANOVA (design: Region (6 levels, with 3 frontocentral and 3 temporo-parietal ROIs)×Word Category (2 levels: action and object words)) to test for a double dissociation for the representations of action- and object-related words, contrasting r values between the corresponding experiential sub-models and the brain activity patterns in the abovementioned ROIs between word groups.

## Results

### Behavioural results

To evaluate the relationship between experiential semantic properties and the six semantic word categories, results of the behavioural semantic rating experiment were evaluated. Mean values and standard errors for both semantic properties, for which word groups differed, and psycholinguistic variables, for which they were matched, are summarised in Table 1.

### Semantic similarities revealed by three linguistic models

The discriminability of semantic word categories in each model RDM was analysed by comparing (t-test) the within-category dissimilarity values for each semantic type with the between-category similarity values. The resulting t-values were larger for the combination of experiential ratings and distributional semantics (t = 6.30; *p* < 0.0001) than for each individual model of experiential information (t = 5.40; *p* < 0.0001) and distributional semantics (t = 4.50; *p* < 0.0001). This suggests that the combined model of integrative experiential and distributional information produces better semantic category discrimination than each of the two measures alone on the level of purely cognitive-linguistic classification.

For both distributional and experiential models, the within-category dissimilarities were lower (M = 0.75 for Distributional; M = 0.26 for Experiential) than the between-category dissimilarities (M = 0.80 for Distributional; M = 0.62 for Experiential), as revealed by a Wilcoxon rank sum test (Z = −3.08, *p* = .002 for Distributional; Z = −3.23, *p* = .001 for Experiential). The integrative model combining distributional and sensorimotor dissimilarities showed a similar pattern (M = 0.51 within-category; M = 0.66 between-category; Z = −6.15, *p* < .001), i.e. the difference between the within/between category dissimilarities (D = 0.16) was larger than that for the distributional model (D = 0.05) but smaller than that of the experiential model (D = 0.26).

### Results from whole-brain RSA searchlights

**Figure 2.**
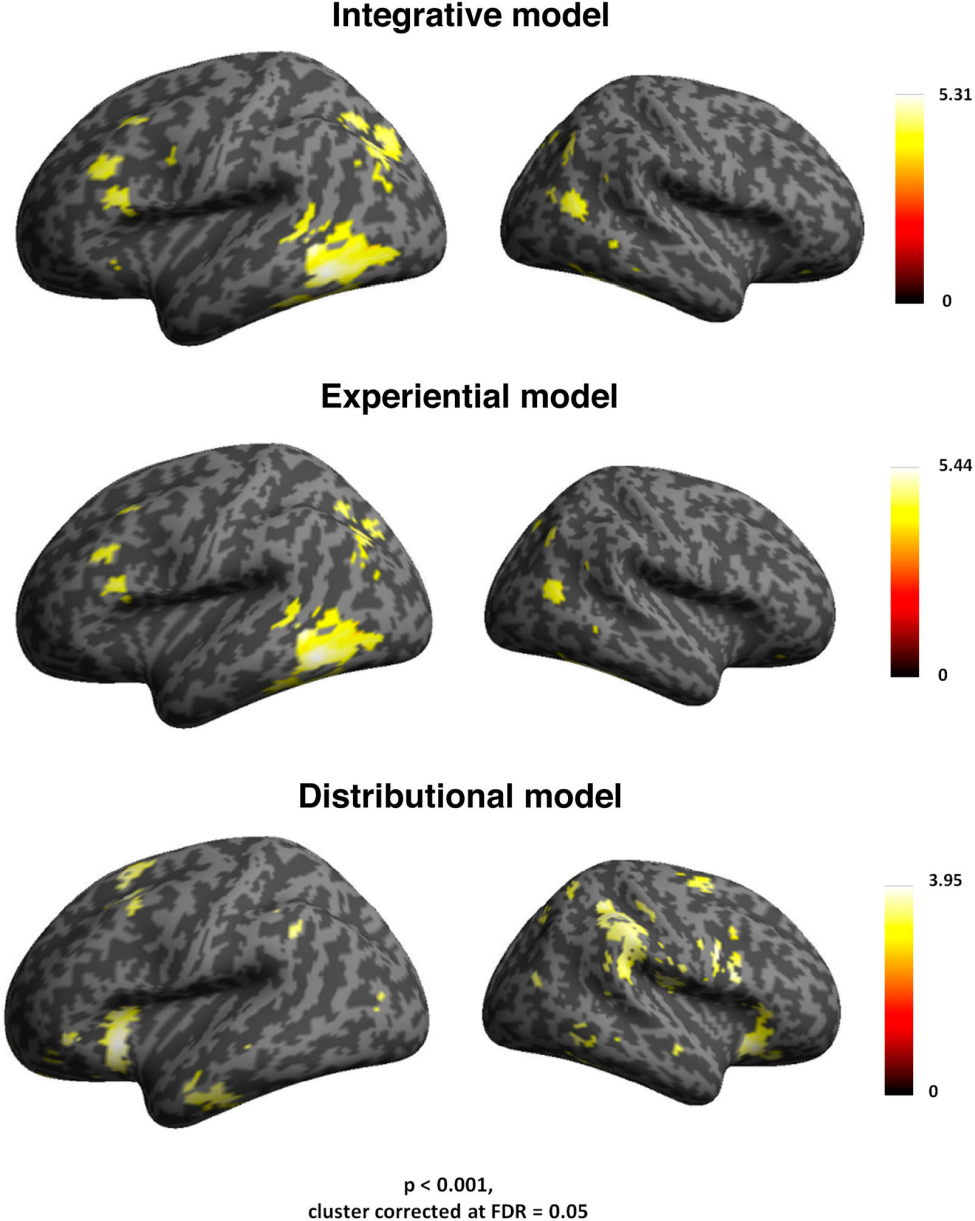
Results from whole-brain searchlight RSA (on the left). Left top panel: widespread activity triggered by the integrative model RDM in distributed prefrontal, premotor, inferior temporal and parieto-occipital cortex. Left middle panel: comparable correlational effects of the experiential model in the pars opercularis of the inferior frontal cortex (BA 44), premotor, inferior temporal and parietal cortex. Left bottom panel: activity triggered by the distributional model RDM in higher-order regions in left inferior frontal cortex (BA 47) and anterior temporal cortex. Results from model comparisons for the whole-brain searchlight RSA (on the right). Right top panel: correlation differences between the brain activity RDMs correlating with integrative model RDM minus the brain activity RDMs correlating with experiential model RDM in left posterior ITG and MTG. Right bottom panel: correlation differences between brain activity RDMs correlating with the integrative model RDM minus the brain activity RDMs correlating with distributional model RDM were seen in dorsal pre-supplementary motor area, and right AG.

Whole-brain RSA searchlights revealed that the integrative model correlated with the similarity structure of the brain activity patterns in fronto-temporal and parieto-occipital cortex (see Table 2 and the top panel of Figure 2). A major cluster of activity was identified in left pITG and FuG, followed by a second one in left occipital lobe, and AG. A third cluster was seen in the pars orbitalis (BA 47), pars triangularis (BA 45) and pars opercularis of the LIFG (BA 44), extending to premotor cortex.

**Table 2.** Results from searchlight RSA for the integrative model. Table of coordinates and significance voxel-level peak values (*p*) in each activation cluster that was correlated with the integrated semantic model integrating distributional and sensorimotor information.

				Coordinates		
Regions	Cluster Extent	Voxel-level *P*	Pseudo *T*	*x*	*y*	*z*
Left fusiform	2850	0.017	4.2	−39	−46	−24
Left Inferior Temporal		0.017	4.45	−51	−46	−12
Left Mid Occipital		0.017	4.31	−24	−64	−36
Left Mid Frontal	1021	0.017	4.68	−33	23	48
Left Insula		0.018	4.14	−30	23	−1
Left Inferior Frontal Opercularis		0.026	3.77	−51	11	29
Left Precentral Gyrus	120	0.030	3.60	−45	11	32
Right Middle Temporal	1514	0.017	4.52	51	−64	10
Right Middle Temporal		0.018	4.23	45	−73	21
Right Angular		0.018	4.20	36	−70	44
Right Inferior Frontal Orbitalis	1604	0.019	4.27	33	32	−12
Right Inferior Frontal Opercularis		0.019	4.02	51	11	25
Right Inferior Frontal Triangularis		0.019	3.74	48	26	2

Additional significant correlations were found in the right MTG, and, to a lesser extent, in right parieto-occipital regions. The grounded experiential related to the brain activity patterns in the same regions as the integrative model, except the LIFG BA 47 (see Table 3 and Figure 2, middle panel).

**Table 3.** Results from searchlight RSA for the experiential. Table of coordinates and significance voxel-level peak values (*p*) in each activation cluster that was correlated with the sensorimotor semantic model.

				*Coordinates*		
Regions	*Cluster Extent*	*Voxel-level P*	*Pseudo T*	*x*	*y*	*z*
Left Inferior Temporal	694	0.016	5.44	−51	−46	−12
Left fusiform		0.016	4.45	−39	−46	−24
Left Mid Temporal		0.016	3.60	−48	−40	4
Left Occipital	312	0.017	4.27	−33	−70	40
Left Angular		0.026	2.97	−30	−55	−31
Left Inferior Frontal Triangularis	153	0.016	4.13	−48	26	21
Left Inferior Frontal Opercularis		0.017	4.03	−48	10	29
Left Precentral		0.017	4.52	−48	14	31
Left Mid Frontal	45	0.027	4.05	−33	23	48
Right Middle Temporal	196	0.018	4.23	51	−64	10
Right Middle Temporal		0.018	4.20	45	−73	21

Semantic similarity mapping of the distributional model led to pronounced correlations with the neural pattern in more anterior fronto-temporal regions, including bilateral IFG (BA 47), bilateral dorsal pre-supplementary motor area (pre-SMA), and AG, which showed pronounced correlations with the model in the right hemisphere (see Table 4 and bottom panel of Figure 2).

**Table 4.** Results from searchlight RSA for the distributional model. Table of coordinates and significance voxel-level peak values (*p*) in each activation cluster that was correlated with the distributional semantic model.

				*Coordinates*		
Regions	*Cluster Extent*	*Voxel-level P*	*Pseudo T*	*x*	*y*	*z*
Left Inferior Frontal (BA 47)	581	0.0051	6.38	−21	23	−20
Right Middle Frontal Gyrus		0.0051	6.23	30	50	25
Left Inferior Parietal (BA 40)	629	0.0051	4.73	−48	−49	48
Left Angular (BA 39)	190	0.0051	4.44	−39	−70	40
Right Precuneus	90	0.0051	5.12	15	−73	44
Left Middle Temporal (BA 22)	518	0.0051	3.52	−57	−64	−24
Left Precentral		0.0051	3.32	−54	5	29
Right Inferior Frontal (BA 47)	581	0.0051	5.27	51	38	−5

When the activity correlated with the experiential model was subtracted from the activity correlated with the integrative model (Integrative minus Experiential: Table 5, Figure 2; right top panel), we found correlation differences in left posterior ITG and MTG.

**Table 5.** RSA results from model comparisons in the searchlight framework. Table of coordinates and significance voxel-level peak values (*p*) in each activation cluster that was correlated with the integrative model contrasted against the distributional semantic model.

				*Coordinates*		
Regions	*Cluster Extent*	*Voxel-level P*	*Pseudo T*	*x*	*y*	*z*
Left Inferior Temporal	320	0.019	2.98	−51	−46	−12
Left Mid Temporal		0.019	2.30	−39	−46	−24

In turn, when the activity correlated with the distributional model was subtracted from the activity correlated with the integrative model (Integrative minus distributional: Table 6; Figure 2, right bottom panel), we found correlation differences in dorsal pre-supplementary motor area, and right AG.

**Table 6.** RSA results from model comparisons in the searchlight framework. Table of coordinates and significance voxel-level peak values (*p*) in each activation cluster that was correlated with the integrative model contrasted against the experiential model.

				*Coordinates*		
Regions	*Cluster Extent*	*Voxel-level P*	*Pseudo T*	*x*	*y*	*z*
Left Mid Frontal	138	0.024	2.98	−33	23	48
Right Angular	200	0.024	2.60	36	−70	44

### RSA results per ROI for all words

**Figure 3.**
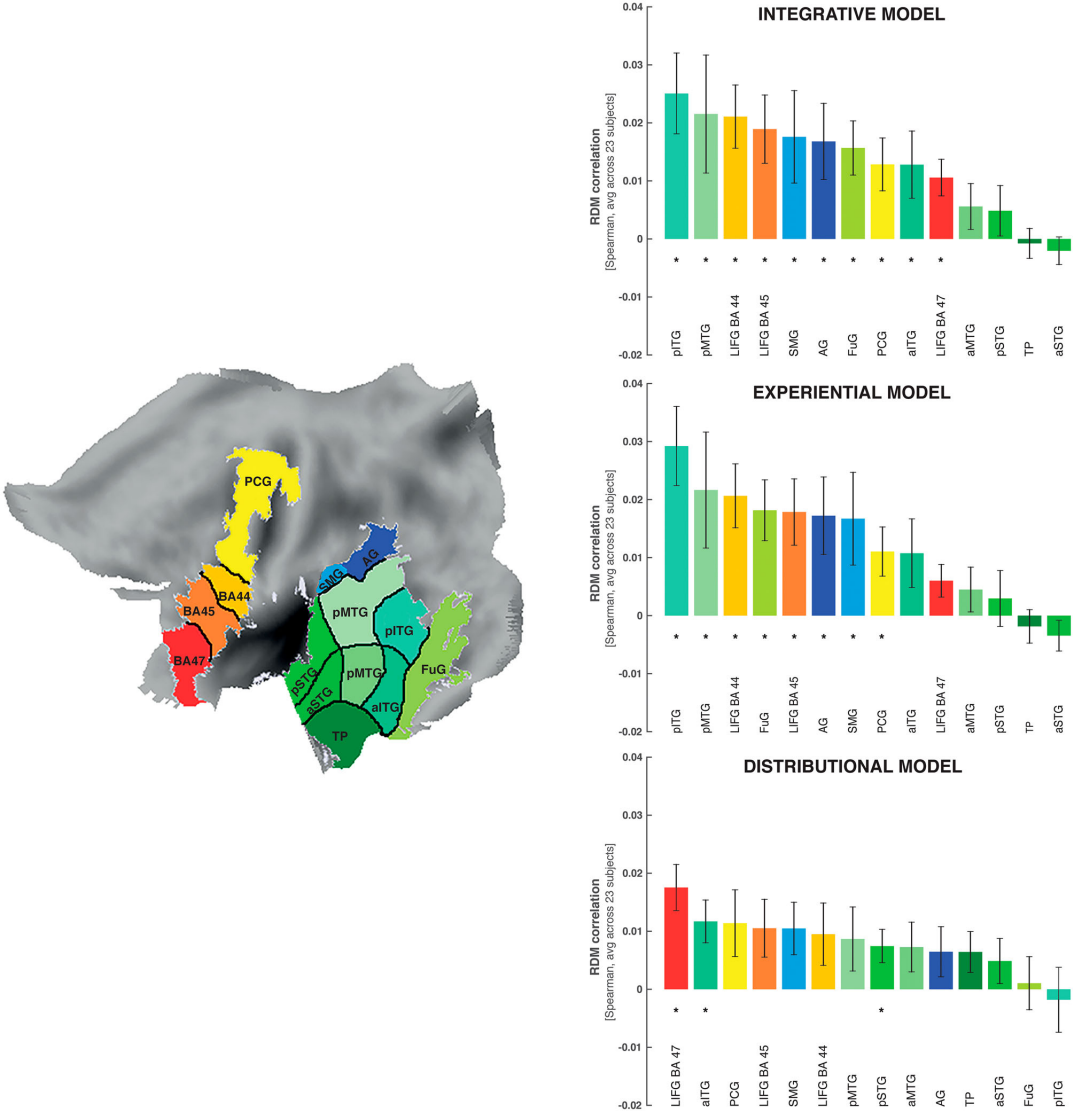
RSA results in the selected ROIs projected onto a flat map of the left hemisphere (the left panel). On the right: The bar graph depicts the averaged model-fMRI pattern correlations for each of the ROI under examination (the corresponding bar is indicated with the same colour as the ROI) across subjects (23). Spearman's rank correlations were calculated to assess the relatedness between brain activity and model RDMs and statistical inference was applied on the single subject correlations using a one-sided signed-rank test across subjects, testing whether the resulting correlation coefficients were significantly greater than zero. Below each bar, the significance value for the test is reported, corrected for multiple testing across brain regions by applying the FDR procedure (marked with an asterisk); the expected FDR was less than 5% (Benjamini & Hochberg, 1995). The horizontal bars in black indicate significant differences from model comparisons after FDR correction across ROIs (FDR = 0.05). Left panel: Effects specific to the LIFG (BA 44), where the distributional model differed significantly from both the sensorimotor and integrative models. Left panel: Effects specific to the left pITG. There was a significant difference (FDR = 0.05) between the effects of the sensorimotor and integrative models and the ones triggered by the distributional model.

The three models produced significant correlations between semantic and brain activity differences in 3 (distributional), 7 (experiential) and 9 (integrative model) areas (see bar diagrams in Figure 3). Therefore, the integrative model returned significant correlations in a greater number of ROIs than the other two models, producing significant semantic similarity effects in pITG-MTG and LIFG (BA 44-45) with additional significant effects in left angular gyrus, precentral, anterior temporal cortex and fusiform gyrus (see Table 7, Figure 3, right top).

**Table 7.** RSA results from ROI analyses testing for relatedness between models and brain activity patterns for all words. Table of correlation values (*r*) and significance values (*P*) between the brain activity patterns in ROIs and the three models for all words per ROI. Correlations values which survive FDR correction for multiple comparisons and the model are indicated by asterisk (*).

		Integrative model			Experiential			Distributional model	
ROI	mean r-val	*p*-val	sig	mean r-val	*p*-value		mean r-val	*p*-value	sig
LIFG BA44	0.021	0.000319	**	0.021	0.000175	**	0.009	0.067067	
LIFG BA45	0.019	0.000127	**	0.018	0.001363	**	0.011	0.020763	
LIFG BA47	0.011	0.001363	**	0.006	0.037435		0.018	7.63E-05	**
PCG	0.013	0.003726	*	0.011	0.006764	*	0.011	0.052271	
TP	−0.001	0.600114		−0.0002	0.699477		0.006	0.032518	
aITG	0.013	0.032518	*	0.011	0.059323		0.012	0.003355	*
aMTG	0.006	0.157277		0.004	0.196536		0.007	0.084762	
aSTG	−0.002	0.7774		−0.003	0.811739		0.005	0.055713	
pITG	0.025	9.07E-05	**	0.029	5.33E-05	**	−0.002	0.117004	
pMTG	0.022	0.006764	*	0.022	0.001726	**	0.009	0.123375	
pSTG	0.005	0.172342		0.003	0.365694		0.007	0.006764	*
FuG	0.016	0.001208	**	0.018	0.001726	**	0.001	0.446606	
SMG	0.018	0.016335	*	0.017	0.020763	*	0.01	0.024221	
AG	0.017	0.012711	*	0.017	0.006147	*	0.006	0.061209	

Likewise, the grounded experiential revealed significant effects across subjects in pITG-MTG, LIFG BA44-45, and angular gyrus (Table 7, Figure 3, right middle) and thus in more areas than the distributional model. The distributional model led to significant effects only in LIFG, and anterior ITG (Table 7, Figure 3, right bottom).

### Results from the distributional statistical model comparisons respectively reveal distributional- and experiential-specific similarity in LIFG (BA 47) and pITG

**Figure 4.**
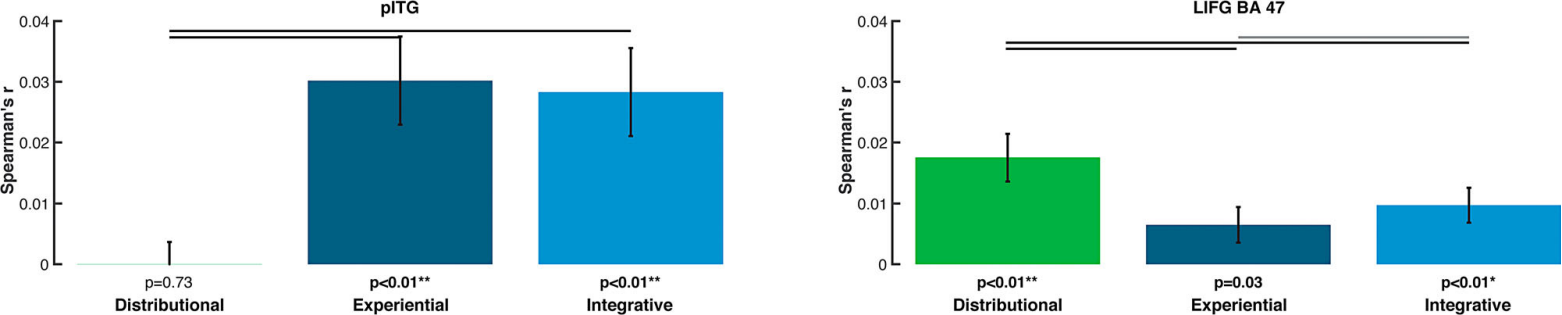
RSA results from model comparisons across all words for selected ROIs and for each of the three models: distributional (green), experiential (dark blue), and integrative model (light blue) (lines above bars indicating significance, *p* < 0.05 FDR corrected). Left panel: Semantic similarity effects specific to the left pITG, where the sensorimotor and integrative models matched the brain activity patterns significantly better than the distributional model. Right panel: Semantic similarity effects specific to the LIFG (BA 47), where the distributional model fits the brain activity patterns significantly better than the sensorimotor and the integrative models. The diagrams are based on the Spearman's rank correlations calculated to assess the relatedness between brain activity and model RDMs.

As shown in Figure 4, the brain activation similarities correlated differently with the semantic similarities obtained from the 3 models. In left pITG, we found significantly higher correlations for integrative and experiential models, as compared with distributional ones, whereas the opposite result was achieved for LIFG. Only significant differences are reported.

### Double dissociation of action and object words in motor and temporo-parietal regions

Contrasting the experiential and the distributional sub-models for action and object words separately revealed category-specific differences between the two semantic types. For action concepts (see Table 8), both experiential and the distributional models correlated with the patterns of activity in LIFG (BA 44-45-47), and left posterior middle temporal cortex.

**Table 8.** RSA results from ROI analyses testing for relatedness between models and brain activity patterns for action words. Table of correlation values (*r*) and significance values (*P*) between the brain activity patterns in ROIs and the three models for object words per ROI. Correlations values which survive FDR correction for multiple comparisons and the model are indicated by asterisk (*).

	Integrative model			Experiential			Distributional model		
ROI	mn(r)	*p*-value	sig	mn(r)	*p*-value	sig	mn(r)	*p*-value	sig
LIFG BA 44	0.0297	0.0008	**	0.0242	0.0107	*	0.0185	0.0374	
LIFG BA 45	0.0255	0.0027	**	0.0212	0.0107	*	0.0168	0.0459	
LIFG BA 47	0.0205	0.0027	**	0.0154	0.0374		0.0140	0.0303	
PCG	0.0163	0.0281		0.0127	0.0755		0.0106	0.0557	
TP	0.0081	0.1234		0.0042	0.1113		0.0080	0.1501	
aITG	0.0099	0.1001		0.0076	0.3110		0.0017	0.3110	
pITG	0.0310	0.0019	**	0.0272	0.0037	*	0.0151	0.0401	
aMTG	0.0067	0.1363		0.0027	0.3434		0.0032	0.1723	
pMTG	0.0222	0.0034	**	0.0149	0.0242		0.0202	0.0208	
aSTG	0.0042	0.2410		−0.0003	0.4822		0.0099	0.1001	
pSTG	0.0046	0.1965		0.0022	0.2800		0.0042	0.6675	
TP	0.0081	0.1234		0.0042	0.1113		0.0080	0.1501	
FuG	0.0137	0.0401		0.0089	0.0801		0.0120	0.0490	
SMG	0.0194	0.0082	*	0.0163	0.0046	*	0.0148	0.1723	
AG	0.0164	0.0593		0.0149	0.8353		0.0036	0.3545	

**Figure 5.**
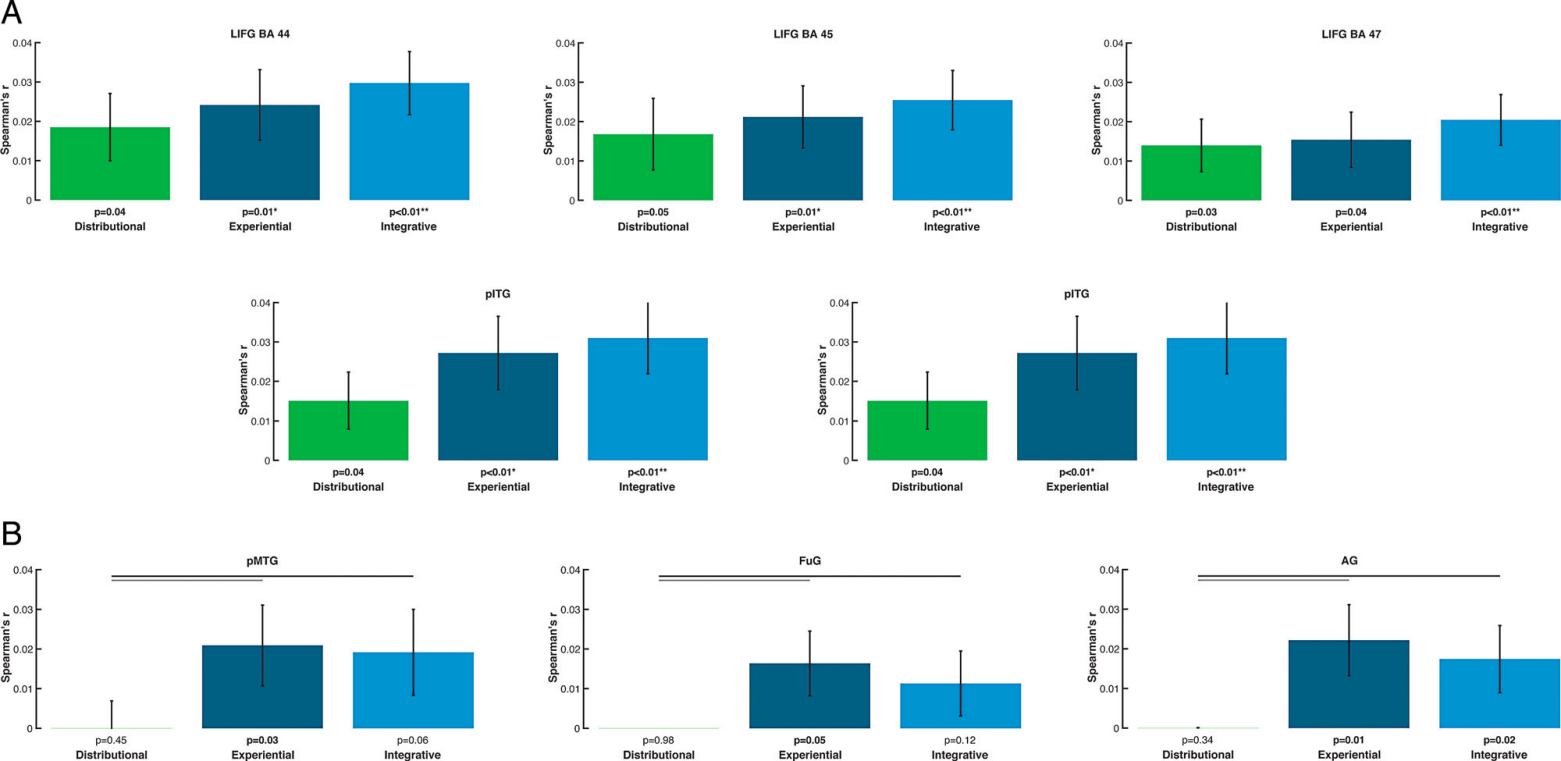
Category-specific results for object- and action-related concepts from model comparisons. The bar graphs depict the averaged correlations between the brain activity RDMs in selected ROIs and the (48 × 48) RDMs for the distributional (green), the experiential (dark blue), and the integrative (light blue) models for the sub-spaces of action and object words. Significant differences are indicated by the horizontal bars in black after FDR correction across models (FDR = 0.05). (A) For action words, there was no significant difference in model performance, as the three models performed equally for LIFG (BA 44-45-47); similar results were seen in pITG and pMTG. (B) For object words, the integrative and experiential models were significantly superior to the distributional model in the pMTG, FuG, and AG.

The integrative model performed comparably to both models, and also revealed a significant correlation with the brain activity patterns in PCG (Figure 5(A)). In contrast, for object concepts (see Table 9), the experiential model performed better than the distributional model in a network of regions comprising the LIFG (BA 44-45), left pITG, left FuG, and left AG.

**Table 9.** RSA results from ROI analyses testing for relatedness between models and brain activity patterns for object words. Table of correlation values (*r*) and significance values (*P*) between the brain activity patterns in ROIs and the three models for object words per ROI. Correlations values which survive FDR correction for multiple comparisons and the model are indicated by asterisk (*).

	Integrative model			Experiential			Distributional model		
ROI	mn(r)	*p*-value	sig	mn(r)	*p*-value	sig	mn(r)	*p*-value	sig
LIFG BA 44	0.0117	0.1113		0.0162	0.0801		−0.0107	0.8828	
LIFG BA 45	0.0122	0.1234		0.0180	0.0848		−0.0163	0.9776	
LIFG BA 47	0.0114	0.1363		0.0091	0.2226		0.0052	0.1802	
PCG	0.0133	0.0261		0.0151	0.0303		−0.0030	0.6675	
TP	0.0007	0.7495		0.0003	0.6675		−0.0125	0.9926	
aITG	0.0060	0.2410		0.0051	0.3657		0.0019	0.4584	
pITG	0.0167	0.0557		0.0169	0.0948		−0.0004	0.2317	
aMTG	0.0114	0.0755		0.0099	0.1723		−0.0005	0.5297	
pMTG	0.0192	0.0631		0.0209	0.0303		−0.0031	0.4466	
aSTG	0.0015	0.6890		0.0010	0.5885		−0.0133	0.9949	
pSTG	0.0029	0.3545		0.0005	0.5769		−0.0014	0.3884	
TP	0.0007	0.7495		0.0003	0.6675		−0.0125	0.9926	
FuG	0.0113	0.1234		0.0164	0.0459		−0.0150	0.9776	
SMG	0.0065	0.2050		0.0070	0.1363		−0.0031	0.6890	
AG	0.0174	0.0163		0.0222	0.0061		−0.0078	0.3434	

The integrative model performed similarly well as the experiential, and was significantly superior to the distributional model in the left pMTG, AG, and FuG (Figure 5(B)). This indicates that mapping of semantic similarities of action-related words is relatively robust across distributional and experiential methods, whereas the meaning of object word is relatively better mapped by the experiential method.

Overall, then, this pattern of results confirmed category-specific semantic mapping differences across distributional, experiential, and integrative models in left frontotemporal and inferior parietal cortex. Semantic mappings were generally more robust for models including experiential semantic information about the items. Indeed, the results also suggest that the distributional model captured the similarity for action concepts in frontotemporal and inferior parietal cortex well, but less so those of object concepts. Semantic mappings for object words were present in left pMTG, FuG and AG reflected experiential (and integrative) information.

**Figure 6.**
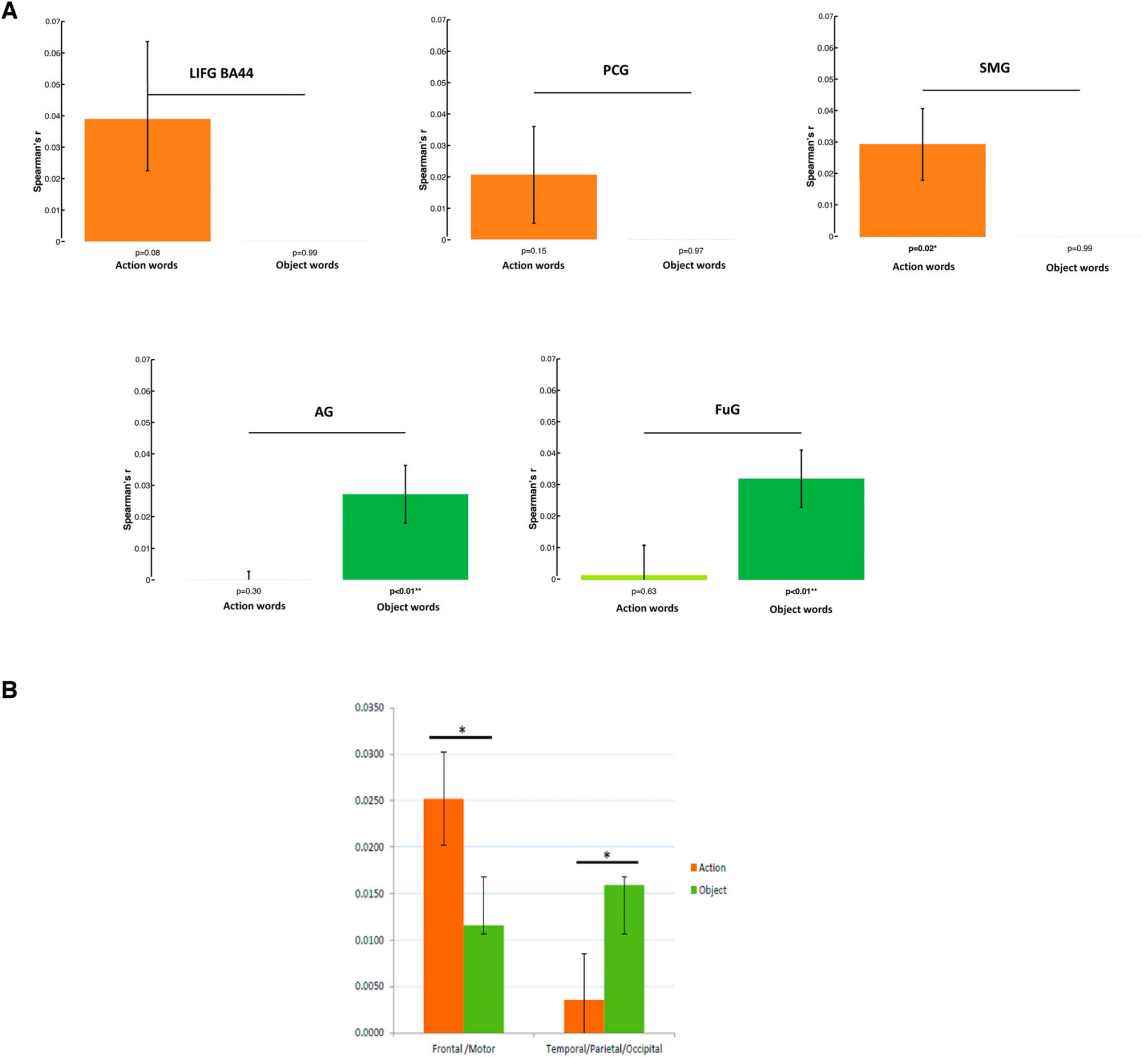
(A) Category-specific results for object- and action-related concepts from model comparisons. The bar graphs depict the averaged correlations between the 48 × 48 brain activity RDMs in specific cortical areas and the model RDMs based on action-related semantic properties (top panels) and visual properties (bottom panel) for the sub-spaces of action (orange) and object (green) words. Significant differences are indicated by the horizontal bars in black after FDR correction across models (FDR = 0.05). Top panel: in LIFG (BA 44), PCG and SMG, the model based on action-related semantic properties was correlated more strongly with the similarity patterns specific to action words as compared to the ones specific to object words. Bottom panel: in the FuG and AG, the opposite was found, i.e. greater correlations between the similarity structure based on the visual properties and the similarity structure of the brain activity patterns for object words than for action words. (B) Results from statistical analyses showing differential mapping of lexicosemantic categories obtained (ANOVA design: Word Category (action verbs vs. object nouns)×Region (frontal vs. temporo-occipital regions of interest)), with a significant cross-over interaction between the factors Region and Word Category (F [1, 22] = 7.45, *p* = 0.01). This effect obtained by employing the integrated semantic vector model revealed a double dissociation in the semantic similarity mappings of action and object words onto focal category-preferential (BA 44, PCG) and category general (SMA, AG, FUG) regions. A comparable interaction effect was also found when the experiential model alone was applied (F [1, 22] = 7.85, *p* = 0.012). The distributional model did not reveal any semantic mapping differences across word categories.

In turn, the direct statistical comparisons between the models of the experiential visual and action-related semantic properties for action and object words revealed a clear differentiation between the underlying representations respectively in the LIFG (BA 44), PCG, and SMG, and in the FuG and AG (see Figure 6(A)). These significantly distinct patterns (FDR = 0.05) indicated a double dissociation between the representation of action and object words based on their respective experiential attributes. These results confirm category-specificity in model-performance differences in motor and higher-order visual regions supporting the comprehension of action and object concepts.

Additional ROI analyses were performed to compare semantic similarities between the large lexicosemantic categories of action-related verbs and object related nouns, as a further sanity check of the data. A statistical analysis of pooled values from category-preferential frontal and posterior areas (see Methods) indicated a differential mapping of lexicosemantic categories (ANOVA design: Word Category (action verbs vs. object nouns)×Region (frontal vs. temporo-occipital)). Please refer to Figure 6(B).

## Discussion

In the present study, we asked how experiential semantic information about the actions and perceptions in which word meaning is grounded and distributional data about word co-occurrences in texts are mapped to distinct neurometabolic activity patterns of the human brain and whether integrating these approaches with each other may lead to improved semantic mappings, including any category discriminability of semantically related action and object concepts. We found that the experiential and integrative models produced semantic mappings in a range of areas, including lateral prefrontal, parietal and middle temporal association cortices. In contrast, the distributional model best correlated with a more focused region in left inferior frontal cortex (BA 47). The integrative model performed better than the distributional model alone in left posterior inferior and middle temporal cortex, and it performed better than the experiential in left supplementary motor area, and right AG.

A further major result concerns the differences in our models’ performance when separately analysing their relatedness to brain activity patterns for the large categories of action and object related concepts and words. Both the experiential and the distributional models, as well as the integrative one, explained the neural similarity structure for action words/concepts (although to a different extent) in LIFG and pMTG, whilst only the experiential and integrative models produced significant correlations with semantic similarities of object words in the pITG, FuG, and AG. Both combined and grounding models showed a double dissociation with selected frontal lobe areas better mapping action semantic features and posterior areas indexing object related perceptual ones. These results suggest that both distributional statistics and grounded experiential data contribute to a reliable decoding of symbol meaning of different semantic types, thus supporting the view that both grounding and distributional information are relevant for the discrimination and representation of concepts (see, for example, Barsalou et al., 2008; Louwerse, 2008). Consistent with earlier work (Fernandino et al., 2022), our results also suggest that grounding information about the actions and perceptions words are used to speak about are generally better reflected in brain response similarities than distributional information about word co-occurrences.

The task applied in the present experiment involved attentive silent reading with occasional typo detection. This task was chosen to ascertain alertness and focused attention to the stimuli. As we were interested in semantic processes elicited by attended-to written stimuli, we avoided a task forcing subjects towards a specific type of semantic processing. Note that a semantic task, such as semantic judgements under a predefined aspect or matching to a target concept or category, would have elicited neurocognitive processes of comparison, which would have overlaid, modulated and thus potentially confounded the process of interest, namely word meaning access. One may still argue that our methodological choice leaves the theoretical possibility that subjects attended to the words but did not understand or semantically process them. However, it is widely believed that skilled readers and users of a language cannot avoid understanding written words they see and attend to. This position is supported by a range of previous neurocognitive studies revealing consistent brain correlates of word and sentence meaning in passive and non-semantic tasks (see, for example, Carota et al., 2012, 2017; Hauk et al., 2004; Pulvermüller et al., 2005; for reviews see Binder & Desai, 2011; Pulvermüller, 2018). Consistent with this claim, the present dataset showed semantic signatures of the word materials that mapped semantic similarities obtained from different linguistic methods. Therefore, the correlation of brain activation similarities with semantic similarities provides clear evidence that the words presented were also understood and their meaning processed. Consistent with earlier work, these results thus show semantic processing occurring independently of the application of a semantic task.

### The importance of experiential information for the representations of concepts

Our present results demonstrate that the grounded experiential and the distributional models predict the representational patterns across different regions considered in the present study. This confirms earlier evidence suggesting that experiential information is representationally predominant over distributional data in multimodal semantic regions (Fernandino et al., 2022). However, the present data further reveal striking differences in model performance when the models are compared within separate regions, as the distributional and experiential models respectively mapped the high-dimensional semantic space in left inferior frontal gyrus (LIFG, BA 47) and in left inferior posterior temporal cortex, in which also the integrative model performed best.

Furthermore, based on both experiential and distributional data, the semantic spaces of action-related verbs and object related nouns could be respectively mapped in specific category-preferential and category-general frontal (motor) and temporo-parietal (visual) areas. In turn, the distributional model complemented these effects, by capturing the brain activity patterns in left frontotemporal regions for action words, but did not match the brain activity patterns specific to object words. Furthermore, these category-specific representational profiles corresponded to the effects seen if the pertinent set of action-related and visually-related properties was selected for model comparison, when a clear double dissociation became manifest in the spatial distribution of their respective representations in category-preferential premotor, and higher-level regions in the ventral and dorsal visual streams in posterior temporal and parietal cortex.

Overall, then, our results from direct measures of metabolic signals during word comprehension support the assumption that the combinatorial knowledge of statistical regularities with which word symbols co-occur in linguistic contexts (e.g. texts or speeches), are not sufficient to form semantic representations of symbol meanings (Andrews et al., 2014; Cangelosi & Harnad, 2001; Harnad, 1990; Searle, 1980; Vigliocco et al., 2004). Rather, a condition sine qua non for a complete picture of the semantic similarity space is the grounding of these symbols in the motor, perceptual, and affective properties of their referents in the “world”, as mediated by dedicated action and perception systems (Barsalou, 2008, 2017; Harnad, 1990; Pulvermüller, 2013, 2018; Searle, 1980). On the basis of earlier work (Cangelosi & Harnad, 2001; Harnad, 1990; Vincent-Lamarre et al., 2016), an integrative model was expected to lead to a better approximation of human semantic similarity judgments as compared to the use of each estimate taken separately. Here though, we found no significant difference in performance on the entire word set as compared to the experiential model, although the numerically larger numbers of areas with significant semantic similarity mappings were greater for the integrative model compared with the other two. The extent to which the explanatory power of integrative models combining distributional and experiential semantics has proven successful for describing the multidimensional semantic space is an empirical issue, and may depend on the linguistic meaning and context under examination. For example, distributional models alone are performing well in decoding sentential meaning, as well as in computing the similarity structure between the semantic content of sentences (e.g. Pereira et al., 2018). Furthermore, successful decoding in sentential contexts could not be achieved based on the experiential semantics of single words alone but requires additional information not explained by compositional semantics (e.g. about the meaning of idioms) for which combinatorial distributional information is essential. A recent study showed that the distributional GloVe model could predict fMRI representational similarities well when applied in isolation, model integration with a model including semantic attributes significantly improved fMRI prediction of sentence meaning (over GloVe alone and attributes alone) in left lateral temporal lobe and left inferior frontal gyrus (also see Anderson et al., 2019, 2020, 2021). Therefore, language-based computational models may come to the fore for predicting multi-faceted concepts/memories (as described by sentences), whereas they may be less valuable for isolated word-related concepts (current study and especially Fernandino et al., 2022). Although the extent to which distributional and experiential models are successful for describing the multidimensional semantic space is an inherently empirical issue, their explanatory power may depend on the type of linguistic meaning and the semantic context under examination, and may increase for semantic contexts beyond single words. Integrating experiential attributes with distributional data is also beneficial for predicting autobiographical event imagination fMRI data (using GloVe models constructed from participants offline descriptions of what they imagined) (Anderson et al., 2020). One reason is that sentential contexts may constrain distributional statistics to the computation of the words that they actually contain (first-order co-occurrence) for estimating semantic similarity, thus reducing the need to exploit second-order co-occurrence in *similar* contexts, as is the case for single words (as performed here exploiting a large corpus of sentences, paragraphs, etc.).

As mentioned, the present study is not the first to apply the distributional semantic model in RSA of brain signals to words (e.g. Anderson et al., 2019; Carota et al., 2017; Fernandino et al., 2022; Liuzzi et al., 2020; Mirman et al., 2017; Pereira et al., 2018; Wang et al., 2020; Xu et al., 2018). In our earlier studies, we used the classical approach of latent semantic analysis (Landauer & Dumais, 1997; see Carota et al., 2017), and a more state-of-art word embedding model (Word2Vec: Mikolov et al., 2013; see Carota et al., 2021), whereas the more recent GloVe method (Pennington et al., 2014) was applied here. When comparing the results, there is surprising consistency regarding anterior inferior frontal cortex, where the highest correlations between semantic and activation similarities were obtained. In addition, consistent similarity mappings were found in parietal cortex (AG). The GloVe method also led to additional significant similarity mapping in dorsal premotor and anterior temporal areas (see Figure 2). In contrast to our first study (Carota et al., 2017), where a range of inferior frontal areas (BA 44, 45, 47) revealed the clearest distributional semantic mapping correlates, the more posterior areas (44, 45) just missed the significance threshold in the present analysis.

A clear performance difference between distributional and grounded models was seen in the analyses of the action vs. object word analyses, where both models performed equally well in frontal areas, but only the grounded model succeeded in similarity mapping in posterior temporal cortex. The reason why, for our data set, the GloVe distributional model explained the similarity structure of the brain activity patterns for action words better than the ones for object words (where sometimes negative RDM correlation values were observed) might be found in the richer argument structure of action words (verbs) as compared to object nouns, that is, the lexical representation of the lexical arguments they take, such as other verbs, nouns and nominalizations, adjectives, and even prepositions, which provides specific information not only about the syntactic expression of the items that can be realised but also about their semantics (e.g. the verb “drink” requiring two noun phrases, one related to a living being and the other to a liquid). However, this suggestion should be addressed in future experiments. Still, these considerations lead to an important caveat concerning our results on category-specificity from the comparison of action and object concepts based on GloVe. Because the performance of different types of distributional language models varies due to the particular type of techniques their metrics rely on (Anderson et al., 2021; Pereira et al., 2018), it is possible that even if different distributional models still differentiate between semantic categories, they may capture category-specific patterns to different extent depending on how much contextual information they take into account, and how. It is also possible that other models are better optimised for explaining object concept similarity. For instance, recent neuroimaging evidence suggested that topological network models may outperform both cooccurrence and experientials in language-specific regions when object-related words are considered (Fu et al., 2022). In particular, Fu et al. (2022) evaluated the performance of different models to assess task-invariant neural representations. The authors employed brain imaging fMRI data from two different tasks, involving picture naming and word familiarity judgements of written words. They constructed one model based on complex network (graph) path relations, with words as nodes and their simple co-occurrences as edges, and proposed that when language is represented as a graph, rich topological properties (node and edge layout patterns) can be computed, capturing information that affects semantic knowledge and language learning. They assessed the performance of the network graph topological models (graph-common-neighbours and graph-shortest-path), of a standard word embedding model such as Word2Vec (Mikolov et al., 2013), and of a model based on simple (edge) co-occurrence. They found that the distributional (Word2Vec) model correlated with activity in left occipital and posterior MTG when the brain imaging data from the picture naming task were examined. The same model correlated with activity in a greater number of cortical regions, also including bilateral anterior temporal lobe when the data from the familiarity judgement were included. However, a conjunction analysis testing for task-invariant effects by assessing the overlapping regions that showed significant positive correlations between neural activity and models showed effects (no surviving voxels). The study of Fu et al. (2022) employed a partial correlation approach regressing out of each language model potential confounds such as visual co-occurrence features, and sensorimotor information. The results bring novel insights on the potential relevance of the (so far little tested) network graph topological models (describing edges and nodes arrangements in a network) to the semantic similarity structure of object-related words. However, important methodological differences regarding the type of tasks, the stimulus set (object nouns), the language (Chinese), the models themselves, none of which was based on the GloVe model employed here, make it difficult to establish a comparison with our present data. Also, these interesting results require further validation in the context of other word semantic classes, such as action verbs. Concerning the results from the RSA analyses, in our present study, our main semantic similarity effect in posterior middle/inferior temporal cortex was linked to the experiential model. However, the corresponding sensorimotor model was only used as a control model for partial correlation analyses, and not compared with the brain activity patterns on its own. Furthermore, no direct comparison of the models was performed, as we did in our present study. However, it is interesting to note that Fu et al. (2022) report task-invariant effects of the network graph topological model just in the two regions highlighted in our present results: the LIFG and the left posterior ITG/MTG. Therefore, despite the abovementioned methodological differences, our present results match the ones by Fu and colleagues in the loci of semantic similarity effects, but also diverge from those results because the semantic similarity mapping in the LIFG and in posterior inferior/middle temporal regions emphasises distributional and experiential information, respectively.

### Hierarchical architecture of the representational semantic systems: category-general semantic similarity mapping in higher-level association cortex

The present results highlight a hierarchical architecture of the representational systems encoding lexical meaning, according to which distributional and grounded experiential semantic properties of word symbols are represented in partly dissociable sets of category-general and category-specific regions in motor and sensory cortex. Our data are consistent with models positing the co-presence of multimodal semantic properties in several higher-order convergence zones holding conjunctive representations derived from multiple low-level sensory and motor representations (Binder & Desai, 2011; Damasio, 1989a, 1989b; Meyer & Damasio, 2009; Pulvermüller 2013, 2021; Pulvermüller et al., 2009; Reilly et al., 2016). Indeed, we found that the LIFG and the pITG respectively best reflected similarity in distributional and experiential properties of the stimulus words. These areas had been found active to written words from a range of different semantic categories, as shown by earlier univariate fMRI studies and likewise by applying k-means cluster analysis (Pulvermüller et al., 2009). Interestingly, these observations are also in line with results from connectivity analyses showing that a cortical network comprising left inferior frontal, and posterior inferior temporal, and inferior parietal cortex supports semantic processing functions specifically (e.g. Xiang et al., 2010). In particular, posterior inferior temporal cortex is known to store lexical information about conceptual features in memory (Carlson et al., 2014; Coutanche et al., 2016; Devereux et al., 2013; Ghio et al., 2016; Hagoort, 2019; Mitchell and Cusack, 2016; Mitchell et al., 2008; Tyler et al., 2013; Clarke and Tyler, 2014), thus supporting a semantic route to reading (e.g. Price, 2000).

Neuronal populations coding for representations of lexical-semantic similarities in the posterior temporal memory circuit may communicate via long-distance connections with population codes in inferior frontal regions, especially in the LIFG (see Fuster, 1997; Pulvermüller, 1999, 2018), as recently confirmed across imaging modalities (e.g. Lau et al., 2008; Pulvermüller, 2013; Xiang et al., 2010). Strengthened activity in this network may arise as a result of the spread of activation from the LIFG.

In turn, the more anterior/ventral aspect of the left inferior frontal cortex is critical for unification of semantic information into linguistic structures (e.g. Hagoort, 2015), a process which operates on combinatorial semantic information and which may require access to the representation of statistical knowledge, such as the one captured by our distributional model. The present results fit this picture well, stressing the importance of the LIFG (BA 47) in semantic processing highlighted in previous literature (e.g. Poldrack et al., 1999; Thompson-Schill et al., 1997), and particularly in the encoding of distributional representations, a finding confirming earlier results (Carota et al., 2017, 2021).

Also, anterior temporal regions showed a relatively stronger correlation of activity patterns with the distributional semantic model, which however did not reach a significant difference compared with the other two models, and did not stand out from the other language regions. In conclusion, the present study confirms a role of the anterior temporal regions in the encoding of semantic similarity, but failed to confirm this region as a possible apex for all forms of semantic knowledge to be brought together, as expected on the basis of the “Hub-and-spokes” model (Hoffman et al., 2018; Lambon Ralph et al., 2017; Patterson et al., 2007), highlighting its contribution within a widespread network of distributed language representations.

### Category-specific representations of action-related and visually-related words

A major finding of the present study was that a sub-network of inferior parietal (AG) and fusiform (FuG) regions reflected object-specific representations of visually-related semantic similarity. Previous fMRI studies have shown that the preferential response of different portions of the FuG to different object categories, for example animals and tools as employed here (Chao et al., 1999; Forseth et al., 2018; Martin, 2007; Tyler et al., 2013; Warrington & Shallice, 1984). Such categorical differentiation has been proposed to arise from higher and lower levels of shared features across the members of different categories of objects, giving origin to category structure (Tyler et al., 2013). It is interesting that, in the present data, the AG may become co-activated with an object-specific FuG region in the ventral visual pathway to code for an intermediate and more specialised level of object-specific representation

The AG encodes cross-modally integrated representations of semantic knowledge (e.g. Fernandino et al., 2015, 2016), as well as category-general representations of statistical knowledge about object words, as our present results show. This area may thus be a multimodal association region binding together different formats and modalities of semantic representations to enable the comprehension of complex conceptual information about object concepts. For instance, earlier evidence suggests that the AG supports the representation of complex object concepts (e.g. plaid jacket), based on simple conceptual constituents (e.g. jacket and plaid) (Price et al., 2015). The semantically coherent representation of these conceptual combinations presupposes the multimodal integration of cross-modal attributes in this well-established semantic “hub” area (Binder et al., 2009; Binder & Desai, 2011; Fernandino et al., 2015, 2022).

In turn, we here found that the posterior ITG/MTG reflected experiential and integrative similarities, but not distributional codes for the set of object words, whilst all models predicted the brain activity patterns for action words in posterior MTG. These differences are consistent with a role taken by this region in storing experiential information, and particularly action-relatedness (Carota et al., 2017, 2021), and action semantics (Hauk et al., 2008). The greater importance of the pMTG in coding for distributional similarities among action verbs as compared to object concepts is also consistent with the it responds specifically to verbs (Elli et al., 2019).

Other studies also found sensitivity of the posterior ITG/MTG to the semantic similarity for a variety of object categories, including tools, when using distributional metrics (Word2Vec) and visual features, including shape (Liuzzi et al., 2020). Discrepancies between these results and our present research outcomes can depend not only on the distributional model employed (see discussion above), but also, more obviously, on the definition of the ROIs in terms of size and locus. For instance, the MTG is more sensitive to action-related tool nouns than nouns referring to animals (Carota et al., 2012, 2017; Desai et al., 2009; Fernandino et al., 2015; Hauk et al., 2008; Martin et al., 1996; Pillon & d'Honincthun, 2011; Vannuscorps & Pillon, 2011). Furthermore, responses to object nouns are more posterior and inferior relative to the ones specific to verbs (Bedny & Caramazza, 2011; Perini et al., 2014), and this can explain differences in the semantic similarity effects reported for object words across recent studies in these regions. Finally, also the use of different distributional methods for generating semantic vectors (e.g.: Word2Vec vs. GloVe) could underlie these differences.

In the current data, it was noteworthy that specialised sub-networks encode distinctive information supporting the disambiguation and identification of specific categories of words and concepts. The additional results from the comparisons of the models coding for specific visual and motor properties clearly further demonstrate the neurocognitive distinctiveness, and distinction, of action and object words. Our present findings thus point to a genuinely experiential semantic origin of category-specific representations: classifying an object and the word used to name it as a member of a category may require simple conjunction of experiential attributes, which confines activity to category-specific temporo-parietal regions.

The semantic sub-space in left motor, inferior frontal (LIFG BA 44), motor, and the SMG was, in turn, well mapped by the model coding for rated action-related properties of the words, consistent with earlier findings on the role of these regions in action semantics (e.g. Damasio & Tranel, 1993; Davey et al., 2015; Dreyer et al., 2020; Grossman et al., 2008; Hauk et al., 2004; Hillis et al., 2004, 2006; Kemmerer et al., 2012; Kemmerer & Tranel, 2003; Pulvermüller et al., 2005; Rueschmeyer et al., 2010; Vukovic & Shtyrov, 2019). Interestingly, these same regions reflected both distributional and experiential data as well.

#### The need for integration of distributional data with grounded experiential semantics

On the basis of earlier work (Cangelosi & Harnad, 2001; Harnad, 1990; Vincent-Lamarre et al., 2016), an integrative model was expected to lead to a better approximation of human semantic similarity judgments as compared to the use of each estimate taken separately. Here though, we found no significant difference in performance as compared to the experiential model.

Our results support the theoretical assumption that the distributional information resulting from the computational manipulation of symbols alone is insufficient for making those symbols interpretable (Andrews et al., 2014; Cangelosi & Harnad, 2001; Harnad, 1990; Searle, 1980; Vigliocco et al., 2004). As postulated by the well-known Searlean Chinese room example, a computer could manipulate strings of Chinese symbols so well to perform as convincingly as a Chinese speaker would, even passing the Turing test, yet it would not understand their meaning. As this would require knowledge of what these symbols are used to communicate about (Harnad, 1990; Searle, 1980). Rather, the grounding of arbitrary symbolic word forms in action and perception knowledge and corresponding brain systems holding this information, at least for some of the symbols (“grounding kernel”: Cangelosi & Harnad, 2001), is essential for building the referential links between the arbitrary and symbolic word forms and the actions, objects and entities in the external world they are used to talk about (Barsalou, 2008, 2017; Harnad, 1990, 2012; Pulvermüller, 2013, 2018; Searle, 1980). The present data are consistent with the view that both linguistic context and the action and object context, in which concepts are experienced since the early stages of language acquisition, critically contribute to coherent word meaning representations (Wittgenstein, 1953/2009).

## Conclusions

We showed that distributional and experiential similarities between concrete written words is reflected by the similarities between word-elicited graded activation patterns in partly distinct networks of frontal and temporo-parietal regions. The resulting neurocognitive picture informs semantic theories suggesting that a representationally complete characterisation of the complexity of lexical meaning cannot be based on distributional information alone, but requires experiential knowledge about the grounding of symbol meaning in action and perception. In the present data, the conjunction of a limited amount of specific experiential properties, such as the dominant action-related and visual attributes of action and object words, was sufficient for mapping the semantic spaces of the corresponding categories in category-specific motor and visual cortex, as well as multimodal connector hub areas, thus being consistent with the relevance of category-specific as well as category-general semantic grounding information (Binder et al., 2016; Fernandino et al., 2016, 2022; Pulvermüller et al., 2009). Still, distributional information contributed to the differentiation between categories as well, drawing upon slightly different and sometimes overlapping association areas (e.g. left inferior frontal cortex). Semantic similarity-mappings of the categories of action and object words were equally successful in frontal cortex with both grounded experiential and distributional models, whereas only the former model succeeded in similarity mapping of object words in temporal cortex. Further work is needed for determining to what extent the different sources of semantic information (e.g. distributional and experiential properties of words, and their interplay, as employed here) contribute to the mapping of the representational content of language in relevant brain networks for different types of concepts, and beyond single words (e.g. sentence, discourse, naturalistic contexts).
